# Inducing Ferroptosis: Sensitization Strategy for Radiotherapy and Its Application

**DOI:** 10.3390/antiox15020237

**Published:** 2026-02-11

**Authors:** Xun Chen, Shangwu Chen, Dongsheng Yu, Wei Zhao

**Affiliations:** 1 Guangdong Provincial Key Laboratory of Stomatology, Guanghua School of Stomatology, Hospital of Stomatology, Sun Yat-sen University, Guangzhou 510055, China; chenx978@mail.sysu.edu.cn; 2 Guangdong Key Laboratory of Pharmaceutical Functional Genes, State Key Laboratory of Biocontrol, Department of Biochemistry, School of Life Sciences, Sun Yat-sen University, Guangzhou 510275, China; lsschshw@mail.sysu.edu.cn

**Keywords:** ferroptosis, radiosensitization, radiotherapy, reactive oxygen species, lipid peroxidation, redox homeostasis

## Abstract

Ferroptosis is a novel regulated cell death caused by the accumulation of iron-dependent ROS and excessive local lipid peroxides in the membrane, widely involved in various physiological and pathological processes. Ferroptosis has emerged as a key mechanism in radiotherapy response. Radiotherapy is an effective treatment for many types of cancers, which not only causes double-stranded DNA break-induced apoptosis, but also induces the production of ROS, leading to oxidative stress and tumor cell death. Recent studies have shown that ionizing radiation in radiotherapy can induce ferroptosis in tumor cells. The combination of radiotherapy and ferroptosis induction can synergistically induce ferroptosis to enhance the sensitivity of tumor cells to radiotherapy, making ferroptosis induction a promising radiosensitization strategy. In this review, we summarize the characteristics and regulation of ferroptosis, analyze the mechanism of radiotherapy-induced ferroptosis, and specifically discuss the different strategies of inducing ferroptosis for radiosensitization. We also point out the shortcomings, future prospects, and research directions of this strategy.

## 1. Introduction

Ferroptosis is a form of regulated cell death first reported in 2012, caused by the accumulation of iron-dependent reactive oxygen species (ROS) and excessive local lipid peroxides in the membrane [1]. Therefore, the ferroptotic process involves the accumulation of Fe^2+^, the generation of free radicals, dysfunction of the antioxidant system, and peroxidation of polyunsaturated fatty acids (PUFAs) [2]. PUFAs can be oxidized through enzymatic reactions and non-enzymatic autoxidation driven by the iron and Fenton reaction. Membrane phospholipids containing PUFAs (PUFAs-PL) are oxidized beyond a certain threshold, leading to plasma membrane rupture and cell death. Ferroptotic cells exhibit typical morphological changes distinct from other forms of RCD, characterized by dysmorphic small mitochondria with condensed membranes and reduced crista [1,3]. So far, it is widely believed that acyl-CoA synthetase long-chain family member 4 (ACSL4) and lysophosphatidylcholine acyltransferase 3 (LPCAT3) [4], which are involved in PUFAs-PL biosynthesis, are the main drivers for ferroptosis, while glutathione peroxidase 4 (GPX4) and solute carrier family 7 member 11 (SLC7A11), which can reduce cellular ROS and peroxides, are the major defenders of ferroptosis. Increasing evidence demonstrates that ferroptosis is widely involved in various physiological and pathological processes, especially in the occurrence, development, and treatment of tumors.

Ferroptosis has emerged as a key mechanism in radiotherapy response. Radiotherapy, as an effective treatment for cancer patients, can not only cause double-stranded DNA break-induced apoptosis, but also induce the production of ROS, leading to oxidative stress and tumor cell death [5]. Ionizing radiation (IR)-induced ROS during radiotherapy can lead to lipid peroxidation and subsequent ferroptosis in cancer cells [6,7]. The mechanism of IR-induced ferroptosis is multifaceted and not fully understood. In theory, as long as it can promote ROS generation, PUFA synthesis, and lipid peroxidation, it will ultimately enhance ferroptosis. Currently, it is believed that radiotherapy-mediated ferroptosis may be attributed to the accumulation of ROS, depletion of glutathione (GSH), upregulation of ACSL4, downregulation of SLC7A11, and other metabolic and immunological mechanisms [6,8,9]. Many studies, including those of cell lines, mouse xenograft tumors, human patient-derived xenograft models, and cancer patients, support that the cell death response to radiotherapy is partially caused by IR-induced ferroptosis [6,7,8]. Ferroptosis has been recognized as one of the important pathways for IR-induced tumor cell death [10].

Given that ferroptosis is one of the cell death modes induced by radiotherapy, inducing ferroptosis can be used to improve radiosensitivity. The combination of radiotherapy and ferroptosis induction can synergistically induce ferroptosis, which will undoubtedly be of great benefit to radiotherapy. In fact, ferroptosis induction plays an important role in making tumor cells sensitive to radiation and improving the efficacy of radiotherapy. Targeting key ferroptosis inhibitors such as GPX4 and SLC7A11 through genetic or pharmacological methods has radiosensitizing effects [6,7,8,11,12]. The application of novel radiosensitizers to induce ferroptosis has become a promising strategy for radiosensitization [13,14,15].

## 2. Mechanism and Regulation of Ferroptosis

**Figure 1:**
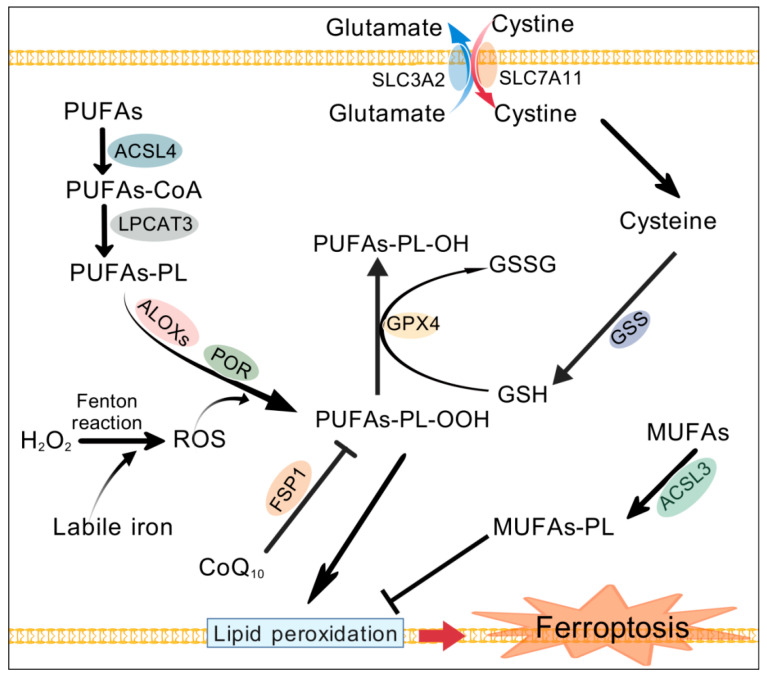
Mechanism and regulation of ferroptosis. The peroxidation of polyunsaturated fatty acids in membrane phospholipids damages the structural integrity of the membrane, leading to ferroptotic cell death. ACSL4 and LPCAT3 catalyze the acylation and acyl transfer of polyunsaturated fatty acids, thereby promoting the biosynthesis of PUFAs-PL, which provides substrates for peroxidation. Labile iron promotes Fenton reaction-mediated ROS production, which, together with ROS generated by enzymatic reactions (ALOXs and POR), promotes peroxidation of PUFAs. GPX4 can eliminate peroxides by using GSH as a reducing agent. The system Xc^−^ imports cystine into cells and supplements the substrate cysteine for GSH synthesis. MUFAs lack a bis-allylic position and are less prone to peroxidation. Correspondingly, MUFAs-PL can prevent ferroptosis. CoQ_10_ cooperates with FSP1 to prevent lipid peroxidation mediated by lipid peroxyl radicals. ACSL3, acyl-CoA synthetase long-chain family member 3; ACSL4, acyl-CoA synthetase long-chain family member 4; ALOXs, arachidonate lipoxygenases; CoQ_10_, coenzyme Q_10_; FSP1, ferroptosis suppressor protein 1; GPX4, glutathione peroxidase 4; GSH, glutathione; GSS, glutathione synthetase; GSSG, oxidized glutathione; LPCAT3, lysophosphatidylcholine acyltransferase 3; MUFAs, monounsaturated fatty acids; MUFAs-PL, phospholipids containing MUFAs; POR, P450 oxidoreductase; PUFAs, polyunsaturated fatty acids; PUFAs-PL, phospholipid containing PUFAs; PUFAs-PL-OOH, lipid peroxides; PUFAs-PL-OH, lipid alcohols; ROS, reactive oxygen species; SLC3A2, solute carrier family 3 member 2; SLC7A11, solute carrier family 7 member 11. Created with BioGDP.com “https://biogdp.com/ (accessed on 15 July 2025)” [24].

Ferroptosis is an iron-dependent form of regulated cell death characterized by the lethal accumulation of lipid peroxides on cellular membranes [1,16]. Regulated cell death (RCD) refers to the form of cell death that can be regulated by various biomacromolecules. Unlike other classical RCD involving specific effector proteins of cell death, it is currently known that ferroptosis is not caused by executioner proteins. On the contrary, the key event of ferroptosis onset is the peroxidation of PUFAs-PL (Figure 1) [17]. Lipid peroxidation refers to the formation of peroxyl radicals on PUFAs, followed by the formation of lipid peroxides.

During the ferroptotic process, PUFAs are mainly oxidized through two mechanisms: enzymatic reactions and non-enzymatic autoxidation driven by the Fenton reaction, with iron playing a prominent role. Arachidonate lipoxygenase (ALOX) family enzymes directly introduce molecular oxygen into PUFAs and PUFA-containing lipids within the membrane [18], while NADPH-cytochrome P450 reductase (POR) provides electrons to P450 enzymes, facilitating the peroxidation of PUFAs [19,20]. PUFAs-PE can also be oxidized to toxic malondialdehyde and 4-hydroxynonenal, which can further react with biomacromolecules [21]. The accumulation of lethal membrane-localized lipid peroxides ultimately leads to cell death. In fact, it is not yet fully understood how lipid peroxides damage cell membranes and result in cell death. The current models include pore formation, membrane phase separation, and the activation of ion channels leading to osmotic lysis. The end stage of ferroptotic cell death involves the permeabilization of the plasma membrane through the formation of pores a few nanometers in radius [22]. Membrane lipid peroxidation can also drive liquid–liquid phase separation and interfere with the structure and composition of membrane rafts [23]. Due to the use of cell-derived giant plasma membrane vesicles as a model in this study, in vivo experiments may be required for validation.

Ferroptosis can be enhanced by a ferroptotic promoter. Due to the fact that the ferroptotic process is triggered by lipid peroxidation, the synergistic interaction of ROS accumulation, lipid provisioning, and the activation of lipid peroxidation enzymes can help induce ferroptosis or enhance ferroptosis sensitivity [25]. PUFAs that are prone to peroxidation, such as arachidonic acid (AA), are first activated by ACSL4 to form acyl-CoA, which then provides its acyl group through LPCAT3 to form phosphatidylethanolamines (PEs) [17,26,27]. Therefore, PUFAs, ACSL4, and LPCAT3 play a crucial role in promoting ferroptosis due to their involvement in the biosynthesis of PUFAs-PL (Figure 1) [26,28]. Given the importance of fatty acid synthesis in ferroptosis, particularly the acetyl-CoA carboxylase (ACC)-mediated synthesis of malonyl-CoA, the significance of ACC in inducing ferroptosis also deserves attention [29]. For example, the activation of ACC promotes IR-mediated ferroptosis, which may be due to the increased production of PUFAs [30] or disruption of iron homeostasis [31]. The activation of ACC drives ferroptosis by disrupting iron homeostasis, indicating a unique coupling between iron homeostasis and lipogenic signaling [31]. However, studies have also shown that the inhibition of ACC can promote ferroptosis under certain conditions [32,33]. Long noncoding RNA (lncRNA) CRCMSL suppresses ACC activity to promote ferroptosis and reduce membrane fluidity by reducing the biosynthesis of MUFAs. The main ACC-catalyzed products are MUFAs, although the carbon chain extension of PUFAs derived from the essential fatty acids linoleate and linolenate requires a carbon source provided by malonyl-CoA. Therefore, the significance of ACC in ferroptosis still needs to be further studied.

Iron is another important promoter of ferroptosis, and controlling iron metabolism will greatly affect the susceptibility of cells to ferroptosis [2]. On the one hand, iron can directly activate lipoxygenases or act as a cofactor for other peroxidizing enzymes. The enzymes that drive ferroptosis, including ALOX, cyclooxygenases (COX), and POR, are all iron-dependent enzymes [19,20,34]. On the other hand, the accumulation of Fe^2+^ in cells forms the labile iron pool (LIP). Labile iron reacts with H_2_O_2_ via the Fenton reaction to generate hydroxyl radicals, which then react with PUFAs-PE to form lipid peroxides [35,36]. It is believed that iron-dependent enzymatic reactions trigger the formation of lipid hydroperoxides, which are substrates for the Fenton reaction that propagate lipid peroxidation [34,37,38]. There are multiple mechanisms for regulating intracellular iron abundance to affect sensitivity to ferroptosis, such as controlling the level of iron-storage protein ferritin through ferritinophagy [38,39].

Due to the fact that lipid peroxides are pathogenic factors in the pathogenesis of ferroptosis, cellular antioxidant mechanisms are the key defense line against ferroptosis, determining the induction or inhibition of ferroptosis. The negative regulation of ferroptosis can be simply categorized as GPX4-dependent and GPX4-independent antioxidant pathways. GPX4 utilizes GSH as an electron donor to catalyze the conversion of toxic lipid peroxides into non-toxic lipid alcohols [40]. Cysteine is the rate limiting substrate for GSH synthesis. It can be produced through the reduction of cystine, which is imported into cells through the cytoplasmic membrane cystine/glutamate antiporter protein (system Xc^−^) composed of the solute carrier family 3 member 2 (SLC3A2) and solute carrier family 7 member 11 (SLC7A11) [41]. Therefore, GPX4, together with GSH and the system Xc^−^ or its SLC7A11 subunit, is crucial for maintaining intracellular redox balance and defending against ferroptosis.

Several GPX4-independent antioxidant mechanisms described below are involved in the defense against ferroptosis. The FSP1-CoQ_10_-NAD(P)H pathway, also known as the radical-trapping antioxidant system, is an independent parallel system that works synergistically with GPX4 to inhibit phospholipid peroxidation and ferroptosis [42]. The inhibition of ferroptosis by ferroptosis suppressor protein 1 (FSP1) is mediated by coenzyme Q_10_ (CoQ_10_, ubiquinone). Reduced CoQ_10_ (ubiquinol) traps lipid peroxyl radicals that mediate lipid peroxidation, while FSP1 acts as a CoQ_10_ oxidoreductase, utilizing NAD(P)H to catalyze the regeneration of CoQ_10_ and restore cellular antioxidant capacity. The inhibition of FSP1 and GPX4 can synergistically induce ferroptosis [42]. FSP1 can also catalyze the conversion of FAD to 6-hydroxy-FAD, which directly inhibits ferroptosis in cells [43]. Similarly, 7-dehydrocholesterol, an intermediate metabolite of distal cholesterol biosynthesis, has been recently presumed to act as a radical-trapping antioxidant [44]. It utilizes the conjugated diene to exert its anti-phospholipid autoxidation function as a natural anti-ferroptotic metabolite.

Unlike PUFAs, monounsaturated fatty acids (MUFAs) are less prone to peroxidation due to the lack of bis-allylic positions. Therefore, the biosynthesis of MUFAs-PL and the displacement of PUFAs by MUFAs in membrane lipids can prevent ferroptosis [45]. The synthesis of MUFAs-PL is mainly regulated by ACSL3 and stearoyl-CoA desaturase 1 [46]. Therefore, the ACSL3-mediated metabolism of MUFAs inhibits ferroptosis [45,47,48].

Nuclear factor erythroid 2-related factor 2 (Nrf2) also affects cell sensitivity to ferroptosis by regulating the expression of antioxidants and iron homeostasis [49]. Under basic conditions, Nrf2 binds to Kelch-like ECH-associated protein 1 (Keap1), leading to its degradation in the proteasome. When responding to oxidative stress, Nrf2 rapidly dissociates from Keap1 and translocates to the nucleus, where it acts as a transcription factor and binds to the Antioxidant Response Element (ARE) in the promoter regions of many antioxidant genes, thereby enhancing their expression [50,51]. For instance, Nrf2 can directly bind to the SLC7A11 promoter region and induce its expression [52]. Therefore, Nrf2 activation promotes radioresistance, in part by upregulating SLC7A11 and inhibiting ferroptosis [52]. Other molecules that regulate SLC7A11 expression, such as p53, can also affect cellular ferroptosis [53].

In summary, the core event of ferroptosis is the lethal accumulation of lipid peroxides on the membrane. The peroxidation of PUFAs-PL mainly involves enzymatic mechanisms mediated by lipoxygenase and non-enzymatic mechanisms driven by iron. PUFAs as peroxidation substrates, enzymes ACSL4 and LPCAT3 that promote PUFAs-PL synthesis, and iron that facilitates peroxidation are key ferroptosis promoters. Ferroptosis suppressors mainly include the GPX4-dependent antioxidant system composed of GPX4, electron donor GSH, and SLC7A11, which imports cystine for GSH synthesis, as well as the radical-trapping antioxidant system represented by the FSP1-CoQ10-NAD(P)H pathway. Contrary to PUFAs, MUFAs are suppressors of ferroptosis. Nrf2 typically defends against ferroptosis through the transcriptional activation of antioxidant genes.

## 3. Radiotherapy-Induced Ferroptosis in Tumor Cells

### 3.1. Ionizing Radiation-Induced Ferroptotic Cell Death

As is well known, radiotherapy is the main treatment for many tumors, and IR-mediated DNA double-strand breaks can induce cell cycle arrest, senescence, and various types of cell death, including apoptosis, necrosis, pyroptosis, autophagy, and mitotic catastrophe [8,54]. In fact, radiotherapy can also promote the production of ROS, leading to the peroxidation of PUFAs [6,55]. Excessive lipid peroxidation can lead to ferroptosis, and the oxidative stress and ferroptosis caused by IR are considered important biological effects in killing tumors [1,10]. Therefore, IR-induced ferroptosis and its significance in radiotherapy have received widespread attention [29,56,57,58,59]. An increasing number of studies have indicated that ferroptosis is an important factor in radiation-induced cell death response, while inhibiting ferroptosis can promote radiotherapy resistance [6,7,8,10].

About five years ago, several studies successively linked ferroptosis with cell death in radiotherapy [6,7,8]. For the first time, research on mouse xenograft tumors has found that IR can induce ferroptosis in tumor cells [6]. The intervention of ferroptosis will alter the efficacy of radiotherapy in murine xenograft models of different tumors and xenograft models derived from human patients [6,7]. The irradiated tumor cells exhibit typical ferroptosis biochemical characteristics and gene expression profiles. The ferroptosis triggered by IR appears independent of the cell type of the tumor, but is enhanced by ferroptosis inducers and partially contributes to IR-induced cell death [7,8]. Moreover, radiotherapy for esophageal cancer patients can also cause ferroptosis, and the level of ferroptosis in patients determines their radiotherapy outcomes and prognosis [8]. Studies on clinical tissues have shown that radiosensitive hepatocellular carcinoma cells exhibit higher levels of ferroptosis, as evidenced by characteristic indicators of ferroptosis such as intracellular lipid peroxidation and Fe^2+^ concentration [60]. So far, it has been documented that radiotherapy can induce ferroptosis in various tumors [61].

### 3.2. Mechanism of Radiotherapy-Induced Ferroptosis

**Figure 2:**
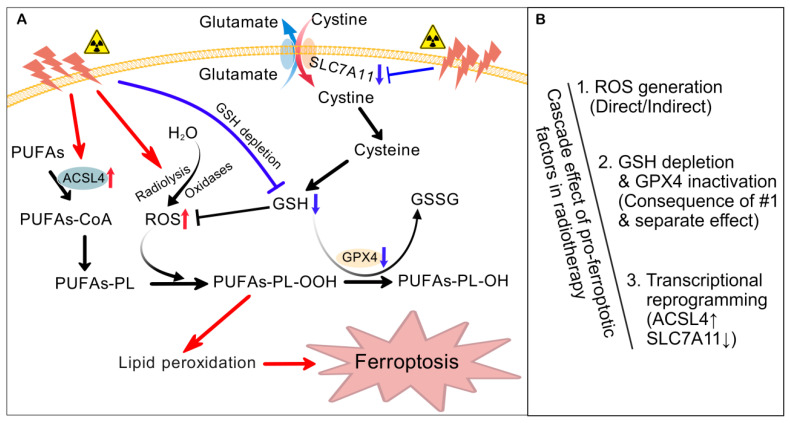
Mechanism of radiotherapy-induced ferroptosis. (**A**) IR induces ferroptosis in radiotherapy. IR can directly increase the ROS generation by promoting water radiolysis and activating cellular oxidases or indirectly reduce consumption of ROS by inhibiting SLC7A11 expression and subsequent GSH synthesis. IR itself can consume and reduce intracellular GSH. High levels of ROS trigger lipid peroxidation. The decreasing GSH impairs the activity of GPX4, leading to the accumulation of lipid peroxides and ferroptosis. IR during radiotherapy can induce the expression of ACSL4 and promote the synthesis of PUFAs-PL, providing the substrates for lipid peroxidation. (**B**) Cascade effect of pro-ferroptotic factors in radiotherapy. IR in radiotherapy mainly promotes ferroptosis through a cascade effect of the three aspects indicated above. ACSL4, acyl-CoA synthetase long-chain family member 4; GPX4, glutathione peroxidase 4; GSH, glutathione; GSSG, oxidized glutathione; PUFAs, polyunsaturated fatty acids; PUFAs-PL, phospholipid containing PUFAs; PUFAs-PL-OOH, lipid peroxides; PUFAs-PL-OH, lipid alcohols; ROS, reactive oxygen species; SLC7A11, solute carrier family 7 member 11. Created with BioGDP.com “https://biogdp.com/ (accessed on 15 July 2025)” [24].

The mechanism of radiotherapy-induced ferroptosis is complex, and the following three aspects may be the most critical (Figure 2). Firstly, IR during radiotherapy can promote the production of ROS [8,62], and high levels of cellular ROS can enhance lipid peroxidation. The generation of ROS may be a direct effect of IR, such as through the radiolysis of cellular water and activation of oxidases [8]. IR also indirectly leads to an excessive accumulation of intracellular ROS by inhibiting the expression of SLC7A11 and the subsequent production of GSH and clearance of ROS [62]. Secondly, IR consumes and reduces the level of GSH in irradiated cells [7,63,64]. The decrease in the GSH level further impairs the antioxidative activity of GPX4, exacerbating the accumulation of lipid ROS and triggering ferroptosis [56,65].

In addition, IR may promote ferroptosis by affecting the expression of many ferroptosis regulatory genes involved in maintaining redox homeostasis or controlling phospholipid oxidation. For instance, the IR-mediated upregulation of ACSL4 expression is believed to enhance ferroptosis during radiotherapy (Figure 2) [8,11,66,67,68]. The deficiency of ACSL4 can significantly reduce radiation-induced ferroptosis and lead to radiation resistance [8]. Some studies have shown that IR itself has an inhibitory effect on the expression of SLC7A11 [6,62]. Radiotherapy may activate the ataxia telangiectasia mutated (ATM) kinase, which inhibits the transcription of SLC7A11 to enhance tumor ferroptosis [6]. There are also reports that IR can induce ferroptosis by inhibiting the expression of GPX4 and SLC7A11 in oral squamous cell carcinoma cells [67] and reducing GPX4 mRNA and GSH levels in intestinal epithelial cells [64]. Several studies have shown that, in the late stage of IR treatment, the expression of SLC7A11 and GPX4 is upregulated in tumor cells [8,11]. It is believed that the upregulation of these ferroptosis inhibiting molecules is considered an adaptive response [8]. Therefore, inactivating SLC7A11 or GPX4 is beneficial for tumor radiotherapy, sensitizing radioresistant cancer cells and xenograft tumors to IR [8,11]. This also provides a theoretical basis for radiotherapy combined with ferroptosis induction through targeting SLC7A11 or GPX4.

In addition to the three main mechanisms mentioned above, other mechanisms may also lead to ferroptosis during radiotherapy. Recently, a metabolic and immunological mechanism has been proposed to explain IR-induced ferroptosis [9]. Radiotherapy facilitates the expression of glutamine transporter protein SLC1A5 and increases glutamine levels in head and neck squamous cell carcinoma (HNSCC). Glutamine blockade combined with radiotherapy can induce immunogenic tumor ferroptosis and improve radiotherapy efficacy. Mechanistically, glutamine blockade enhances the expression of radiotherapy-mediated interferon regulatory factor 1, which increases intracellular Fe^2+^ concentration and induces ferroptosis by upregulating transferrin receptor expression [9]. In addition, the combined treatment enhances CD47 expression and hinders macrophage phagocytosis, thereby weakening the therapeutic effect. Therefore, the dual blockade of glutamine and CD47 can enhance radiotherapy-induced ferroptosis and improve the tumor microenvironment.

Obviously, ferroptosis is a consequence of IR-induced cellular oxidative stress. It is speculated that high levels of ROS weaken the cellular antioxidant system’s defense against ferroptosis, promoting lipid peroxidation (Figure 2). There is currently no evidence to suggest that IR-induced genomic instability or mutations in key ferroptosis regulatory genes directly lead to ferroptosis. This may be one of the key points worth exploring in the future. IR in radiotherapy does indeed induce the expression of ferroptosis promoters such as ACSL4 and COX2 [8,69]. IR also induces the expression of ferroptosis inhibitors, such as SLC7A11 and GPX4, but this is considered an adaptive response [8]. The mechanism and significance of activation or upregulation of these key molecules require further investigation. As mentioned above, IR can induce various types of RCD, including ferroptosis. Therefore, it is necessary to evaluate the contribution of ferroptosis in radiotherapy-induced cell death. Research has found that the inhibition of ferroptosis reduces cell death similarly to the inhibition of apoptosis and necroptosis, and the effect of the ferroptosis inhibitor is even better [8,70]. The results indicate that IR-mediated ferroptotic cell death is an important cell death response in radiotherapy.

The radiation dose and dose rate are key factors in regulating the degree of ferroptosis. Research has shown that radiation-induced ferroptosis is dose-dependent, and different doses may have a significant impact on the intensity of ferroptosis induction. For example, high-dose radiation is more likely to cause intracellular iron overload, ROS burst, and GSH depletion, thereby exacerbating lipid peroxidation and ferroptosis processes [69]. Fractionation can reduce the impact of ferroptosis [70]. However, the specific dose–response relationship and the differences in response of different tissue cell types still need further clarification.

Taken together, IR in radiotherapy induces ferroptotic cell death in cancers by enhancing lipid peroxidation. Radiotherapy can directly increase the production of ROS and the consumption of GSH and can also reduce the synthesis of GSH and weaken the antioxidant function of cells by affecting the expression of ferroptosis regulatory genes. This makes it possible to enhance the sensitivity of radiotherapy by inducing ferroptosis in tumor cells and provides potential targets. Radiotherapy-induced ferroptosis is not only related to cancer cells but also to other cell types in the tumor microenvironment (TME), which will be discussed in a separate section below.

## 4. Radiotherapy-Induced Ferroptosis in the TME

**Figure 3:**
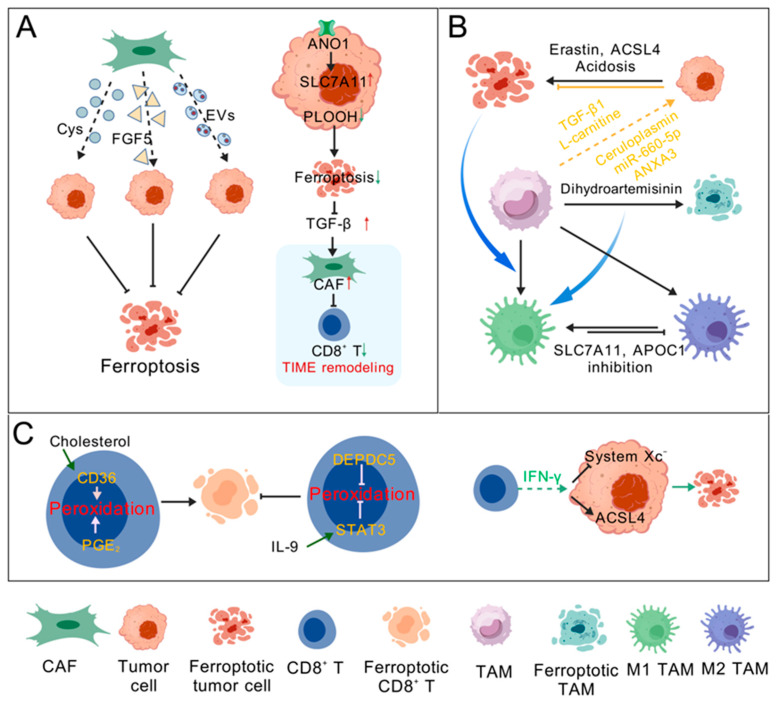
Ferroptosis plays multiple roles in the TME. (**A**) CAFs mediate ferroptosis resistance in tumor cells. CAFs regulate tumor cell ferroptosis within the TME by secreting cysteine, growth factors, or extracellular vesicles. On the contrary, ferroptosis in tumor cells can also affect CAF function and reshape the tumor immune microenvironment. (**B**) Ferroptosis mediates reprogramming of TAMs in the TME. Ferroptosis and related inducers/inhibitors can mediate TAM reprogramming. TAMs, in turn, regulate ferroptosis in cancer cells through paracrine effects and extracellular vesicles. (**C**) CD8^+^ T cells mediate ferroptosis of tumor cells. High cholesterol levels in the TME and PGE_2_ signaling can induce ferroptosis in CD8^+^ T cells. IL-9 activates STAT3, leading to reduced lipid peroxidation in CD8^+^ T cells and resistance to ferroptosis. DEPDC5 protects CD8^+^ T cells from ferroptosis by reducing ROS production and lipid peroxidation. Conversely, anti-tumor CD8^+^ T cells can release IFN-γ through downregulating the system Xc^–^ and stimulating ACSL4 expression, inducing ferroptosis in tumor cells. ACSL4, Acyl-CoA synthetase long-chain family member 4; ANXA3, annexin A3; APOC1, apolipoprotein C1; CAF, cancer-associated fibroblast; Cys, cysteine; DEPDC5, DEP domain containing 5; EVs, extracellular vesicles; FGF5, fibroblast growth factor 5; IL-9, Interleukin 9; PGE_2_, prostaglandin E2; PLOOH, phospholipid hydroperoxide; TAMs tumor-associated macrophage; TIME, tumor immune microenvironment. Created with BioGDP.com “https://biogdp.com/ (accessed on 15 July 2025)” [24].

The crosstalk between immune cells and cancer cells in the TME regulates tumor progression and therapeutic resistance through ferroptosis [71]. Cancer-associated fibroblasts (CAFs) can secrete some metabolites or growth factors, such as cysteine [72] or fibroblast growth factor 5 [73], to promote GSH synthesis or bind to corresponding receptors in tumor cells, thereby inducing tumor cell resistance to ferroptosis or chemotherapy-induced ferroptosis (Figure 3A). CAFs also act on cancer cells by secreting exosomes [74,75,76]. On the contrary, ferroptosis in tumor cells can also affect CAF function. The anoctamin 1 (ANO1)-mediated inhibition of cancer ferroptosis induces the production and release of TGF-β and recruits CAFs into the tumor immune microenvironment (TIME), leading to immunotherapeutic resistance [77]. Ferroptosis and related inducers/inhibitors can mediate the reprogramming of tumor-associated macrophages (TAMs) in the TME (Figure 3B). Tumor cell ferroptosis, mediated by erastin, ACSL4, and acidosis, promotes M1 macrophage polarization [78,79]. Dihydroartemisinin-induced ferroptosis in TAMs also promotes their M1 polarization [80]. Targeting APOC1 or SLC7A11 in TAMs can promote the repolarization of M2 into M1 macrophages [81] or inhibit M2 polarization [82] through the ferroptosis pathway. In addition to the immunostimulatory effects of ferroptosis on macrophages, ferroptotic tumor cells also exhibit ferroptosis-related immunosuppressive effects on TAMs. For example, ferroptotic tumor cells can induce macrophages to secrete pro-inflammatory IL-1β [83] or activate STING signaling in macrophages [84] to promote aggressive tumor growth. TAMs can also induce cancer cell ferroptosis resistance through paracrine signaling, such as with TGF-β1 [85] and L-carnitine [86]. Extracellular vesicles secreted by TAMs or cancer cells can also be used as a mediator to achieve ferroptosis-related cross-talk between tumor cells and macrophages [71]. Similarly, CD8^+^ T cells can also mediate tumor cell ferroptosis (Figure 3C). On the one hand, CD8^+^ T cells have developed multiple mechanisms to evade ferroptosis in the TME [71,87]. Tumor cells have also developed various strategies, such as upregulating SLC7A11 to outperform CD8^+^ T cells in cystine uptake, to induce anti-tumor CD8^+^ T cell ferroptosis and evade their cytotoxic effects [88]. Immunotherapy-activated CD8^+^ T cells, in turn, can release IFN-γ to induce ferroptosis in tumor cells by downregulating SLC3A2 and SLC7A11 [89].

It is known that radiotherapy reprograms the TME and ultimately affects anti-tumor efficacy [90]. The immune system plays an important role in the anti-tumor effect induced by radiotherapy [90]. Due to the involvement of ferroptosis in the regulation of TIME, radiotherapy-induced ferroptosis in the TME is also worthy of attention. Irradiated tumor cell-released microparticles (RT-MPs) reprogram M2 into M1 macrophages in TME, inducing immunogenic death primarily through ferroptosis [91]. T cells, especially CD8^+^ T cells, are required for IR-induced anti-tumor immunity. IR not only increases T cell infiltration but also enhances T cell cytotoxicity. CD8^+^ T cells secrete IFN-γ to induce ferroptosis in cancer cells by stimulating ACSL4 expression [92] or inhibiting SLC7A11 expression [6], thereby sensitizing cancer cells to radiotherapy. The downregulation of SLC7A11 expression, mediated by CD8^+^ T cells activated by immunotherapy or induced by ATM activated by radiotherapy, jointly enhances the ferroptosis of tumor cells, indicating that immunotherapy can synergistically improve tumor radiosensitivity with radiotherapy [6]. On the other hand, radiotherapy may also induce the suppression of anti-tumor immunity, including the recruitment of regulatory T cells, myeloid-derived suppressor cells, and suppressive macrophages [90]. Due to the effects of radiotherapy-induced ferroptosis on TME and the possibility of inducing inhibitory TME through radiotherapy [71,90], we should pay attention to its impact on TME when using ferroptosis induction as an adjuvant therapy.

## 5. Inducing Ferroptosis for Radiosensitization

**Figure 4:**
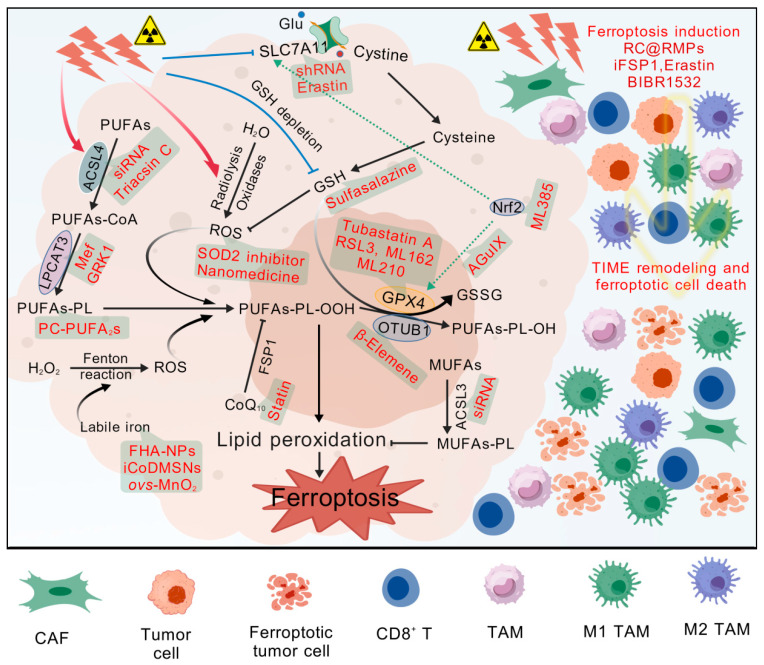
Inducing ferroptosis for radiosensitization. The left panel, with a tumor cell as the background, shows the pathway of IR-induced ferroptosis in tumor cells, as well as the targeting sites or molecules involved in ferroptosis induction presented in red text. Triacsin C via ACSL4, Mef and GRK1 via LPCAT3, and PC-PUFA_2_s regulate the levels of PUFAs-PL. Sulfasalazine can reduce intracellular GSH, while erastin indirectly inhibits GSH synthesis by downregulating SLC7A11. The reduction of GSH and slowdown of its clearance through SOD2 inhibition enhance the accumulation of ROS produced by IR. *ovs*-MnO_2_, and other nanomaterials promote ROS production by increasing labile iron and iron-mediated Fenton reactions. The accumulation of ROS triggers lipid peroxidation, while the inhibition of GPX4 by RSL3 and ML162 inhibitors and GSH depletion-mediated GPX4 inactivation accelerate lipid peroxidation. Radiotherapy combined with ferroptosis induction can enhance ferroptotic cell death and improve the sensitivity of tumor cells to radiotherapy or overcome radioresistance. Meanwhile, as shown on the right panel, ferroptosis induction and radiotherapy-induced ferroptosis can also reshape TIME by reprogramming M2 TAMs into M1 TAMs, increasing CD8^+^ T cell infiltration and cytotoxicity and promoting the secretion of IFN-γ by CD8^+^ T cells, thereby improving radiosensitivity. ACSL3, acyl-CoA synthetase long-chain family member 3; ACSL4, acyl-CoA synthetase long-chain family member 4; CoQ_10_, coenzyme Q_10_; FHA-NPs, iron and hyaluronic acid-based nanoparticles; FSP1, ferroptosis suppressor protein 1; Glu, glutamate; GPX4, glutathione peroxidase 4; GRK1, ginsenoside RK1; GSH, glutathione; GSSG, oxidized glutathione; iCoDMSNs, clustered cobalt nanodots; iFSP1, FSP1 inhibitor; LPCAT3, lysophosphatidylcholine acyltransferase 3; Mef, mefloquine; MUFAs, monounsaturated fatty acids; MUFAs-PL, phospholipids containing MUFAs; Nrf2, nuclear factor erythroid 2-related factor 2; OTUB1, ovarian tumor domain-containing ubiquitin aldehyde binding protein 1; *ovs*-MnO_2_, oxygen-vacancy-rich MnO_2_ nanoflower; PC-PUFA_2_s, phosphatidylcholine with two polyunsaturated fatty acyl tails; PUFAs, polyunsaturated fatty acids; PUFAs-PL, phospholipids containing PUFAs; RC@RMPs, nanomaterial loading RSL3 into irradiated tumor cell-released microparticles; ROS, reactive oxygen species; SLC7A11, solute carrier family 7 member 11. Created with BioGDP.com “https://biogdp.com/ (accessed on 15 July 2025)” [24].

Acquired radioresistance is the main cause of radiotherapy failure [93]. Ferroptosis is one of the important biological effects caused by radiotherapy, making ferroptosis induction a promising radiosensitization strategy. Inducing ferroptosis can be used in various cancer treatment methods to improve efficacy and overcome treatment resistance [13]. There are various strategies for inducing ferroptosis, mainly based on the pathological mechanism of ferroptosis, which can be summarized as follows (Figure 4 and Table 1). Firstly, radiotherapy is combined with direct interference with the expression or activity of key ferroptosis inhibitory proteins [11,12,94]. Secondly, radiosensitization can also be achieved by regulating PUFA metabolism or promoting ROS production and GSH depletion [95,96]. Thirdly, increasing ferroptosis by interfering with the TME is another option for radiosensitization. Finally, new technologies, especially nanomedicine, are employed to induce ferroptosis for radiosensitization [97,98].

**Table 1:** Summary of radiosensitization studies by inducing ferroptosis.

Targets	Mediators	Application	References
SLC7A11, GPX4	Erastin, sulfasalazine, RSL3, liproxstatin-1, ML162, FIN56	Radioresistant lung cancer cells, CDX, PDX; esophageal cancer patients	Lei G et al. [8]
system Xc^−^	Imidazole ketone erastin, sorafenib	CDX of sarcoma, PDX of lung adenocarcinoma and glioma	Ye LF et al. [7]
SLC7A11	Knockdown	NPC cells	Yang F et al. [11]
SLC7A11	p53	Cancer cells and patients	Lei G et al. [99]
SLC7A11	Nrf2, ML385	ESCC	Feng L et al. [52]; Yan L et al. [100]
GPX4	Knockdown, RSL3	CDX of ESCC	Chen C et al. [12]
GPX4	RSL3	Radioresistant NSCLC cells	Ma Y et al. [101]
GPX4	Erastin	Radioresistant NSCLC cells	Pan X et al. [94]
GPX4	β-Elemene	Radioresistant gastric cancer cells	He J et al. [102]
GPX4	HMGA2	Pancreatic cancer cells	Luo Z et al. [103]
GPX4	lncRNA OTUD6B-AS1	Radioresistant colorectal cancer cells	Zhang Z et al. [104]
GPX4	Tubastatin A	Breast cancer cells	Liu S et al. [105]
GPX4	Nanomaterial AGuIX	Triple-negative breast cancer cells	Sun H et al. [106]
GPX4	RRM1	Cancer cells	Gao Y et al. [107]
FSP1	Statin, CoQ	Radioresistant cells	Lin X et al. [108]
ACSL3	shRNA	Radioresistant cells	Cao Y et al. [109]
ACSL4	ASCL4 siRNA	Breast cancer cells	Kwon YS et al. [95]
ACSL4	METTL14	ESCC	Jin Y et al. [110]
ACSL4	LncRNA-RRFERV	NPC patients	Xu Q et al. [111]
ACSL4	PCK2	Tumor-repopulating cells, NPC patients	Li Z et al. [112]
ROS and GSH	Superoxide dismutase 2	NPC cells	Amos A et al. [96]
ROS and GSH	Nanomaterial	Melanoma cells and CDX	Lin Y et al. [98]
ROS and GSH	Nanomaterial	Breast cancer and solid tumor	Shi J et al. [113]; Song H et al. [114]; Wei M et al. [115]
ROS and GSH	Sulfasalazine	Colorectal cancer cells	Kerkhove L et al. [116]
ROS and GSH	Nanomaterial	Breast cancer cells and CDX	Liu Y et al. [97]
Multi-targets	Multifunctional nanomaterial	Triple-negative breast cancer	Zeng L et al. [117]

CDX, cell line-derived xenograft; ESCC, esophageal squamous cell carcinoma; NPC, nasopharyngeal carcinoma; NSCLC, non-small-cell lung cancer; PDX, patient-derived xenograft.

### 5.1. Targeting Key Molecules That Inhibit Ferroptosis

Inhibiting key ferroptosis regulatory molecules such as GPX4 and SLC7A11 can trigger ferroptosis. These inhibitors are called ferroptosis inducers (FINs) and have radiosensitizing effects. There are several aspects that can illustrate the necessity of intervening in these molecules. First, SLC7A11 [118,119] and GPX4 [12,120,121] are frequently upregulated in many cancers. Secondly, as an adaptive response to radiotherapy-induced ferroptosis, cancer cells typically upregulate the expression of GPX4 and SLC7A11 [8,11,105]. Furthermore, cancer cells with acquired radioresistance significantly upregulate the expression of GPX4 and SLC7A11, demonstrating a strong resistance to ferroptosis [60,94,99,122]. Therefore, the genetic or pharmacological inhibition of SLC7A11 [6,7,8,11] or GPX4 [7,12] can enhance radiotherapy-induced ferroptosis and improve tumor radiosensitivity. Moreover, the reactivation of ferroptosis by FINs can also restore the radiosensitivity of radioresistant cancer cells [29,94].

Several effective GPX4 covalent inhibitors have been reported, such as RSL3, ML162, and ML210 (Figure 4). RSL3 and ML162 are the first reported GPX4 inhibitors, belonging to activated alkyl chloride complexes. For example, RSL3 treatment greatly enhances the radiosensitivity of radioresistant non-small-cell lung cancer (NSCLC) cells [101]. But these small molecule inhibitors face some challenges. Firstly, their targets of action remain uncertain. Recent studies have even questioned the authenticity of RSL3 and ML162 as direct inhibitors of GPX4 [123]. Studies have shown that RSL3 sensitizes glioma cells to IR by inhibiting transglutaminase 2-mediated DNA repair and epithelial–mesenchymal transition [124]. RSL3 and ML162 cannot inhibit the enzymatic activity of recombinant GPX4 but are effective inhibitors of another selenoprotein, thioredoxin reductase 1 (TXNRD1) [123]. Although TXNRD1 exhibits a strong and extensive reducing activity parallel to GPX4 in cells, and the inhibition of TXNRD1 can also induce cell death, it has different physiological functions in cells. Therefore, before these compounds are further used in preclinical studies, it may be necessary to reevaluate their true enzyme targets and mechanisms of action to prevent off-target effects and corresponding toxicity and adverse effects [123]. The second challenge is the possibility of these molecules becoming drugs. Due to the lack of drug-like stability and selectivity, their further clinical applications are restricted [15]. Tubastatin A, an inhibitor of histone deacetylase 6 (HDAC6), directly binds to GPX4 and inhibits GPX4 enzymatic activity, regardless of its inhibitory effect on HDAC6. Therefore, Tubastatin A can enhance the anti-tumor effects of radiotherapy by inhibiting GPX4 enzymatic activity to overcome the radioresistance of cancer cells [105]. The side effects caused by its HDAC6 inhibitor need to be explored. Targeting other components in the GPX4 activity axis or indirectly targeting upstream molecules of GPX4 can also sensitize tumor cells to radiotherapy. β-Elemene, a compound derived from the Chinese herb *Curcuma zedoaria* (Wenyujin), promotes ferroptosis and reverses radioresistance in gastric cancer by inhibiting the OTUB1-GPX4 interaction [102]. AGuIX nanoparticles enhance IR-induced ferroptosis in triple-negative breast cancer by targeting the NRF2-GPX4 signaling pathway [106]. These nanoparticles preferentially accumulate at the tumor site and amplify radiation effects, causing nanoscale ionizing damage to tumor cells. Moreover, the small hydrodynamic diameter allows them to be rapidly eliminated through the kidneys. They have entered phase II clinical trials as radiosensitizers for brain metastases. Although these inducers that use GPX4 as an indirect target have potential prospects, the biggest challenge they face is the specificity of their effects.

System Xc^−^ or its subunit SLC7A11 is another critical molecule for the targeted induction of ferroptosis. The knockdown of SLC7A11 with shRNA sensitizes nasopharyngeal carcinoma (NPC) cells to IR [11]. Erastin induces ferroptosis by inhibiting system Xc^−^ and leading to GSH depletion and the indirect inactivation of GPX4 [15]. Therefore, erastin can reduce the radioresistance of NSCLC cells [94]. Due to poor solubility, metabolic instability, and low bioavailability, the clinical application prospects of erastin are worrying [125]. It can also sensitize tumor cells to radiotherapy by indirectly intervening in the upstream regulators of SLC7A11. As is well known, IR triggers the expression of Nrf2, which regulates the transcription of SLC7A11 [14]. Therefore, Nrf2 reduces the radiosensitivity of ESCC by promoting SLC7A11 expression and inhibiting ferroptosis [52]. The inhibition of Nrf2 by a novel Nrf2 inhibitor ML385, in turn, promotes ferroptosis and radiotherapy sensitivity [100]. An important drawback of GPX4 and SLC7A11 as targets is that they are all important functional molecules of cells, and their inhibition may affect the function of normal cells. For example, GPX4 inhibition may induce ferroptotic cell death in immune cells, potentially impairing anti-tumor immunity [126]. Due to the importance of GPX4 function, germline GPX4 deficiency leads to early embryonic lethality [8]. Therefore, it is particularly important to develop new tumor-specific targets that exhibit significant differential expression between tumors and normal tissues.

Studies have found that the upregulation of CoQ synthesis shifts ferroptosis dependence in acquired radioresistance from GPX4 to FSP1, indicating that FSP1 plays a dominant role in the development of radioresistance [108]. The additional administration of statin during radiotherapy, which could hinder CoQ production, effectively resensitizes radioresistant cells to radiation, suggesting that inhibiting the FSP1-CoQ pathway is a promising therapeutic strategy for reversing radioresistance. Oleic acid protects cancer cells from IR-induced ferroptosis caused by the accumulation of PUFAs-PL in an ACSL3-dependent manner [109]. Targeting ACSL3 combined with FIN erastin can synergistically enhances anti-tumor effects in radioresistant tumors [109]. This also makes ACSL3 a potential target for inducing ferroptosis. The significance of these two targets still needs further study, especially in the development of specific inhibitors.

### 5.2. PUFA Metabolism-Mediated Promotion of Ferroptosis

As described above, ACSL4 contributes to the activation of PUFAs and synthesis of PUFAs-PE, making it an important ferroptosis promoter. It has been reported that the expression of ACSL4 is significantly increased in established radioresistant breast cancer cells, and the inhibition of ACSL4 using siRNA or inhibitor triacsin C could overcome the radioresistance [95]. However, this effect is attributed to the inhibition of DNA damage responses and the induction of cell apoptosis through the regulation of FOXM1 by ACSL4 [95]. Due to the important role of the upregulation of ACSL4 expression in radiotherapy-induced ferroptotic cell death [8], it is necessary to carefully evaluate the impact of ACSL4 inhibition on cell ferroptosis and radiotherapy efficacy and develop more specific small-molecule inhibitors. A study on C. elegans has shown that dietary PUFAs can promote ferroptosis [127]. Recent studies have found that phospholipids with two polyunsaturated fatty acyl tails are an important driver of ferroptosis, while dietary phosphatidylcholine with two polyunsaturated fatty acyl tails (PC-PUFA_2_s) promotes ferroptosis in cancer cells [17,128]. Exogenous PC-PUFA_2_s accumulate in mitochondria and bind to Complex I in the electron transport chain, which may lead to an increase in mitochondrial ROS levels. These mitochondrial peroxides further promote their accumulation in the endoplasmic reticulum, ultimately leading to ferroptosis. Therefore, combining radiotherapy with a supplementation of PC-PUFA_2_s or a diet rich in PC-PUFA_2_s may be one of the strategies for improving radiosensitivity. In addition, Mefloquine (Mef), a quinoline ethanol approved for the treatment of malaria infections, enhances the efficacy of anti-PD-1 immunotherapy through LPCAT3-induced tumor ferroptosis [129]. Ginsenoside RK1 (GRK1), an essential component of ginseng, promotes ferroptosis in liver cells by activating the ACSL4/LPCAT3/ALOX5 signaling pathway, thereby alleviating hepatic fibrosis [130]. Obviously, Mef and GRK1 have the potential for radiosensitization, but their role and significance in radiotherapy need further investigation.

### 5.3. Interference with ROS Production and GSH Depletion

Given the important promoting role of ROS and inhibitory effect of GSH in ferroptosis, the additional production of ROS and depletion of cellular GSH will help make tumor cells more sensitive to radiotherapy [56]. For example, the knockdown of superoxide dismutase 2 can induce oxidative stress, making NPC cells sensitive to IR through ferroptosis induction [96]. The radiosensitizing effect depends on the activity of dihydroorotate dehydrogenase (DHODH), indicating that DHODH inhibitors should be used with caution during radiotherapy for NPC patients. A nanomedicine constructed from ferroptosis inducer hemin and thioredoxin 1 inhibitor can promote ROS production through the Fenton reaction and inhibit ROS depletion, significantly improving the sensitivity of ferroptosis in tumor radiotherapy [98].

Several other nanomedicines can effectively deplete intracellular GSH through GSH exhaustion, biosynthetic inhibition, and photodynamic oxidation, thereby improving the efficacy of radiotherapy for breast cancer and overcoming solid tumor radioresistance [113,114,115]. Sulfasalazine is a drug approved by the US Food and Drug Administration for the treatment of rheumatoid arthritis and inflammatory bowel disease, which can enhance the radiosensitivity of hypoxic colorectal cancer cells. The radiosensitizing effect is attributed to the reduction in GSH and thioredoxin reductase levels [116]. Developing new uses for old drugs is relatively more convenient. It must be pointed out that increasing cellular ROS and oxidative stress is a double-edged sword, and its toxic side effects on normal cells must be taken seriously. Targeting tumor cells with relevant drugs through nanomaterials may be one direction.

### 5.4. Radiosensitization Strategies for Inducing Ferroptosis by Targeting TME

As mentioned above, RT-MPs can reverse the tumor inhibitory TME by inducing immunogenic ferroptosis and polarizing M2 to M1 macrophages [91]. On this basis, a therapeutic system has been designed to load the ferroptosis inducer RSL3 into RT-MPs (RC@RMPs) (Figure 4). In the study of a mouse tumor model, RC@RMPs can inhibit tumor growth and enhance the efficacy of anti-PD1 therapy by reprogramming the TME and inducing ferroptosis, indicating that the combination of ferroptosis inducers and radiotherapy has broad prospects in tumor treatment [131]. The inhibition of FSP1 induces ferroptosis. The FSP1 inhibitor (iFSP1) significantly increases immune infiltration, including dendritic cells, macrophages, and T cells, and synergizes with immunotherapy, indicating that FSP1 inhibition represents a new tumor therapeutic strategy [132]. Erastin has been shown to enhance the efficacy of anti-PD-L1 immunotherapy by inhibiting SLC7A11 in TAMs and then reprogramming TAMs, which is believed to synergize with radiotherapy [82,133]. BIBR1532, a telomerase inhibitor, enhances radiotherapy-induced ferroptosis and activates the cGAS-STING pathway to promote the anti-tumor efficacy of radioimmunotherapy [134]. The above studies indicate that reprogramming the TIME or activating anti-tumor immunity can synergistically improve the efficacy of radioimmunotherapy. However, there is still much work to be done for radiosensitization based on targeted TME, such as screening for effective sensitizers and determining their different effects on tumor cells and microenvironmental immune cells. Of course, it is possible to develop a dual-target strategy that not only induces TME-mediated ferroptosis but also directly promotes ferroptosis in tumor cells, which will effectively enhance its role in radiosensitization.

### 5.5. Adoption of New Technology

The development and application of ferroptosis-inducing nanomaterial can further improve the radiosensitizing effect of ferroptosis inducers and increase their clinical application potential [106,115,117,135]. For example, the combination of radiotherapy and iron and hyaluronic acid-based nanoparticles (FHA-NPs) has greatly improved the therapeutic effect of cancer both in vitro and in vivo (Figure 4) [136]. Hyaluronic acid nanoparticles are stable, safe, and non-toxic. Due to the clinical risks associated with increased exogenous iron, such as liver damage, an iron-free, oxygen-vacancy-rich MnO_2_ nanoflower (*ovs*-MnO_2_) has been developed to promote ferroptosis and alter the tumor microenvironment, thereby improving the efficacy of radiotherapy [97]. This nanomaterial induces the release of intracellular free iron (Fe^2+^), which acts as an activator of the Fenton reaction and enhances the accumulation of intracellular ROS, promoting ferroptosis and improving the therapeutic efficacy of radiotherapy. In vitro and in vivo evaluations have shown that *ovs*-MnO_2_ has high biosafety and stability, can accumulate in tumor areas, and has no significant systemic toxicity or adverse effects, demonstrating good application prospects. Clustered cobalt nanodots (iCoDMSNs) induce ferroptosis by upregulating heme oxygenase 1 and expanding the labile iron pool in cancer cells, providing a promising tumor radiosensitizer [135]. Cobalt, as an essential trace element, has a lower toxicity in vivo compared to other nonessential metals. iCoDMSNs have also demonstrated encouraging capabilities in tumor penetration and photoacoustic imaging. Radiotherapy can be combined with ferroptosis induction therapy using nanomaterial to enhance its therapeutic effects on certain malignant tumors, indicating that nanomedicine has broad application prospects in ferroptosis-assisted radiotherapy [136]. Nanomaterials loaded with ferroptosis inducers should have good biocompatibility and stability, without significant toxicity, especially those that can accumulate in tumor areas or specifically target tumor cells. In the future, nanomaterials that can increase intracellular iron content or ROS levels and target them with tumor-specific antibodies may have promising development prospects.

## 6. Main Challenges and Solutions

The main issue with inducing ferroptosis for radiosensitization is the damage to normal tissues. Many studies have shown that inducing ferroptosis can lead to damage to healthy tissues. Erastin induces ferroptosis in rat embryonic cardiomyocytes in vitro [137]. The administration of erastin to healthy mice not only induces ferroptosis, but also induces pathological changes in healthy tissues of mice [138]. In fact, ferroptosis can lead to immune cell death, bone marrow impairment, liver and kidney damage, cachexia, and secondary tumorigenesis, all of which have been well reviewed [25]. In theory, inducing ferroptosis not only promotes the radiosensitivity of cancer cells, but also causes radiotherapy-induced damage in normal tissues [139,140]. For example, ferroptosis plays a role in radiotherapy-induced lung injury [14]. It has been found that ferroptosis is associated with irradiation-induced damage to various normal tissues, such as the intestine, heart, lungs, and skin [141].

The first solution is to determine the vulnerability of cancer cells to ferroptosis and induce them in a targeted manner. Cancer cells are expected to be more sensitive to ferroptosis than normal cells. It is currently known that specific mutations and a loss of function in certain tumor suppressor genes reprogram the lipid metabolism of cancer cells, making them more susceptible to ferroptosis by enhancing PUFA biosynthesis or elevating PUFAs-PL levels, such as retinoblastoma 1 deficiency in prostate cancer [142] and von Hippel-Lindau deficiency in clear cell renal cell carcinoma [143]. Some mutations in these genes may disrupt iron homeostasis and enhance ROS production. For example, synovial sarcoma with malic enzyme 1 deficiency exhibits an accumulation of ROS and labile iron, making it particularly susceptible to ferroptosis [144]. Some oncogenic mutations promote the dependence of cancer cells on anti-ferroptotic factors such as GPX4 and SLC7A11 or cysteine, making them sensitive to ferroptosis. Cancer cells with mutations in the epidermal growth factor receptor or isocitrate dehydrogenase 1 are sensitive to ferroptosis induced by cystine deprivation or the GPX4 inhibitor, respectively [145,146]. In addition to mutations, the metabolic characteristics of certain types of cancer cells, such as mesenchymal features with abundant PUFAs-PL or elevated levels of labile iron pools in therapy-resistant cancer cells, make them susceptible to ferroptosis [147,148,149]. Therefore, more comprehensive studies are necessary to find out ferroptosis vulnerability in different cancers or design a stratification strategy to determine suitable induction regimens. It is necessary to ensure that the combination of ferroptosis induction and radiotherapy has a greater killing effect on tumor cells than on healthy tissues.

Next, the combined use of FLASH radiotherapy (FLASH-RT) and ferroptosis induction may reduce the damage of ferroptosis to healthy tissues [150]. The analysis of the impact of the radiation dose rate on lipid peroxidation has shown that, under conventional dose rate irradiation, the production of lipid peroxides in normal tissues is dose-dependent, while no or a low induction of lipid peroxidation was observed under ultra-high dose rate irradiation in FLASH-RT [151,152]. In other words, FLASH-RT can increase lipid peroxidation and induce ferroptosis in tumor cells, but compared with conventional radiotherapy it does not significantly improve lipid peroxidation and ferroptosis in normal tissues [152]. The changes in lipid peroxidation and ferroptosis may be attributed to the intrinsic differences in iron levels between normal and cancerous tissues [150,152]. Although the exact mechanism behind the protective effect of FLASH-RT on normal tissues is not yet clear, FLASH-RT provides an improved therapeutic window by minimizing damage to normal tissues while maintaining tumor control. This also provides an excellent opportunity for the combination of radiotherapy and ferroptosis induction.

Another obstacle to preventing the application of ferroptosis induction in clinical practice is the availability of appropriate inducers. There are currently no safe FINs that can be considered for clinical use [136]. Due to poor drug selectivity and stability, some FINs are still in the preclinical stage or unable to undergo clinical translation [14,15]. Some approved anti-cancer drugs or other drugs, such as cisplatin [153], sorafenib [154,155], bupivacaine [156], and ketamine [157], have been proven to be ferroptosis inducers. Their significance in radiotherapy and radiosensitization still needs to be reevaluated. Therefore, it is necessary to identify more precise ferroptosis inducers as radiosensitizing agents in the future. Of course, this includes the development of targeted delivery systems for related drugs.

## 7. Conclusions

Radiotherapy is a common cancer treatment method, but its main limitations are radioresistance and damage to normal tissues [136]. Researchers have made significant efforts to improve the efficacy of radiotherapy, overcome radioresistance, and reduce radiation damage. Inducing ferroptosis during radiotherapy is considered a promising radiosensitization strategy. The advantages of combining ferroptosis induction therapy with radiotherapy are obvious, as cancer cells are more susceptible to IR and ferroptosis than normal cells [13,29,49]. Therefore, FINs exhibit significant radiosensitizing effects in cancer cells [13,29,99,140,158], indicating that FINs have good application prospects in radiosensitizing or overcoming radioresistance. In the future, clinical translational research should be accelerated based on existing preclinical studies. It is necessary to further screen tumor-specific ferroptosis induction targets and inducers and develop nano-inducers that specifically target tumor cells through nanotechnology. Exploring new strategies to reduce healthy tissue damage mediated by ferroptosis is also important, which may focus on evaluating the combination of FLASH-RT and ferroptosis induction.

## Data Availability

No new data were created or analyzed in this study. Data sharing is not applicable to this article.
